# A Systematic Review on Recent Trends, Challenges, Privacy and Security Issues of Underwater Internet of Things

**DOI:** 10.3390/s21248262

**Published:** 2021-12-10

**Authors:** Delphin Raj Kesari Mary, Eunbi Ko, Seung-Geun Kim, Sun-Ho Yum, Soo-Young Shin, Soo-Hyun Park

**Affiliations:** 1 Department of Financial Information Security, Kookmin University, Seoul 02707, Korea; delphinraj@kookmin.ac.kr (D.R.K.M.); junsan86@kookmin.ac.kr (S.-H.Y.); 2 College of Computer Science, Kookmin University, Seoul 02707, Korea; sinaa821@kookmin.ac.kr; 3 Ocean System Engineering Research Division, Korea Research Institute of Ships & Ocean Engineering, Daejeon 34103, Korea; sgkim@kriso.re.kr; 4 Special Communication & Convergence Service Research Center, Kookmin University, Seoul 02707, Korea; sy-shin@kookmin.ac.kr

**Keywords:** Underwater Internet of Things (UIoT), trends, challenges, security and privacy

## Abstract

Owing to the hasty growth of communication technologies in the Underwater Internet of Things (UIoT), many researchers and industries focus on enhancing the existing technologies of UIoT systems for developing numerous applications such as oceanography, diver networks monitoring, deep-sea exploration and early warning systems. In a constrained UIoT environment, communication media such as acoustic, infrared (IR), visible light, radiofrequency (RF) and magnet induction (MI) are generally used to transmit information via digitally linked underwater devices. However, each medium has its technical limitations: for example, the acoustic medium has challenges such as narrow-channel bandwidth, low data rate, high cost, etc., and optical medium has challenges such as high absorption, scattering, long-distance data transmission, etc. Moreover, the malicious node can steal the underwater data by employing blackhole attacks, routing attacks, Sybil attacks, etc. Furthermore, due to heavyweight, the existing privacy and security mechanism of the terrestrial internet of things (IoT) cannot be applied directly to UIoT environment. Hence, this paper aims to provide a systematic review of recent trends, applications, communication technologies, challenges, security threats and privacy issues of UIoT system. Additionally, this paper highlights the methods of preventing the technical challenges and security attacks of the UIoT environment. Finally, this systematic review contributes much to the profit of researchers to analyze and improve the performance of services in UIoT applications.

## 1. Introduction

**Figure 1:**
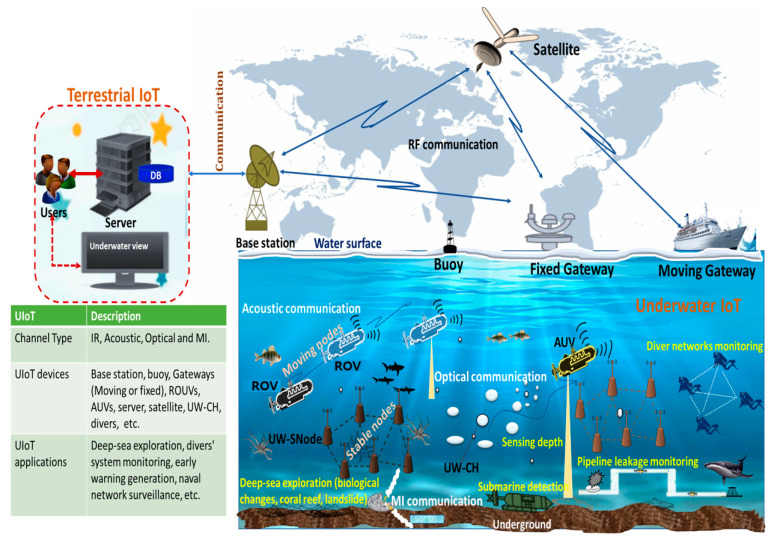
UIoT Architecture.

During the past few decades, researchers and developers have shown much interest in developing UIoT applications such as deep-sea exploration, divers’ system monitoring, early warning generation, naval network surveillance, etc. As shown in Figure 1, the UIoT network consists of heterogeneous devices such as underwater sensor nodes (UW-SNodes), underwater cluster heads (UW-CHs), remotely operated underwater vehicles (ROVs), unmanned underwater vehicles (UUVs), autonomous underwater vehicles (AUVs), etc. The UIoT devices can be fixed or mobile, moving from one location to another to gather information and transmit that information via digitally linked devices in water bodies such as the gateway or buoy in surface water. In addition, other devices like moving gateways, satellites, base stations, etc., are utilized to expand the communication range of UIoT applications.

In the recent survey produced by the United States National Oceanic and Atmospheric Administration (NOAA), 97% of the earth’s surface is covered with water [1]. The UIoT environment is coped with smart sensing underwater devices that are installed with heterogeneous functionalities. Many researchers have proposed different methodologies to design and develop various UIoT applications in the last few years. However, the challenges and limitations are still concerns for the UIoT environment based on the application, channel types and channel characteristics. Channel types define the type of medium used in UIoT environments such as RF, acoustic, optical (VLC: visible light communication or IR: infrared) and MI, and channel characteristics represent the technical factors that affect the medium used in UIoT environments, such as propagation speed, turbulence, pressure, node mobility, etc. [2] Security attacks and privacy issues are the other key challenges in the current UIoT system [3].

This research aims at providing a survey of the state-of-the-art research, communication technologies, challenges, security attacks and privacy issues and provides the mitigation methodology to overcome the challenges and security attacks in the current UIoT system. Furthermore, this research will help the researchers and developers to build new UIoT applications by considering the best channel type with security and privacy models. The key contributions of this paper are briefed under research goals in Table 1.

The layout of this paper is delivered as follows: Section 2 represents the prior study insights and recently used communication technologies of the UIoT system. Section 3 describes the technical challenges, security attacks and privacy issues of UIoT system. Section 4 provides the available methods to overcome the challenges, security attacks and privacy issues of the UIoT system. Section 5 highlights the findings, future work and directions of UIoT system, and Section 6 concludes the paper.

## 2. Q1: What Are the Recent Trends of UIoT System?

This section discusses the recent trends and applications developed in the UIoT system along with the communication technologies of the current UIoT system.

### 2.1. Prior Research

Many articles discuss the latest research and applications developed in the UIoT system [4]. For example, in [5], Gussen et al. unveiled a survey on underwater communication technologies, including the pros and cons of using optical, acoustic and RF channels in the UIoT environment. Furthermore, the research shows that the RF channel is unsuitable for the underwater environment due to its high absorption rate. In [5,6], the channel characteristics of electromagnetic (EM) signals in UIoT and the use of EM signals in the military application were discussed. In [7,8,9,10], the challenges and merits of using acoustic signals in UIoT were discussed. Furthermore, the research shows that an acoustic signal reveals low absorption rates underwater. Therefore, the acoustic signal is used for long-distance communication in the UIoT environment, but the drawbacks are low bandwidth (1–100 kHz), limited speed (≈1500 m/s) and high delay in data transmission. 

In [11,12,13,14], the latest research on underwater optical communication (UwOC) techniques was discussed, and the strength and weaknesses of optical signals were shortened. Additionally, the research showed that UwOC are used for short-range communication with a high data rate in the UIoT environment, but UwOC cannot be applicable for long-range distances due to high attenuation. 

**Figure 2:**
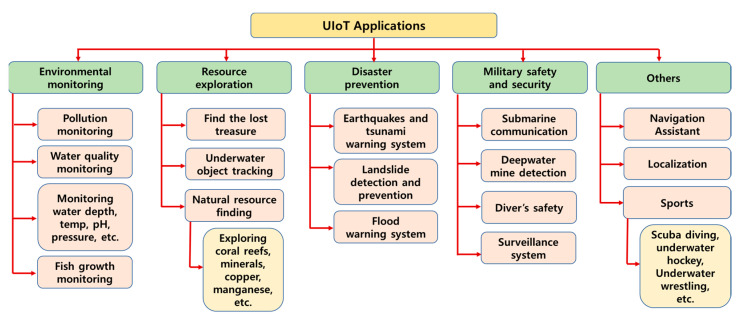
Existing applications of UIoT.

In [15], Kumar et al. developed a single hybrid optical, acoustic modem to achieve a high bandwidth rate, low battery consumption and long-distance data transmission. In [16], a built-in optical, acoustic communication technique was proposed by integrating the optical system into the existing acoustic communication technology to offer a high data rate, long-distance data transfer and low latency in underwater communication. In addition, from [17,18,19,20], other acoustic–optical combined technologies were discussed. In [21], Delphin et al. proposed the new technique by considering multiple mediums and bandwidths based on the distance for reliable data transmission in the UIoT environment. In [22], Delphin et al. developed the underwater hybrid software-defined modem to support the fast and reliable communication system in UIoT. Figure 2 shows that the UIoT applications are grouped into five major categories and have numerous subdivisions according to the survey carried out by Chien-Chi Kao et al. [23]. Moreover, in [24,25], the classifications and descriptions of each UIoT application are indicated. 

### 2.2. Communication Technologies of UIoT

Based on the research highlighted in Section 2.1, the recent communication trends in UIoT are described underneath and the essential channel attributes are briefed in Table 2.

From the physics perception, unlike satellite, TV, mobile and radio communication frequency ranges, the conductivity of radiofrequency in seawater is very high. Thus, Radiofrequency (RF) wave propagation is affected strongly. For this reason, it is not easy to establish links using ultra-high frequency (UHF) and very high frequency (VHF) more than 10 m away from the sea surface. As for lower frequencies, RM attenuation can be considered short enough for reliable communication to occur over a few kilometers. However, the frequencies from 3 kHz to 30 kHz and from 3 Hz to 3 kHz are not enough to transmit at high data rates.

The channel performance and behavior are the main difference between optical and RF channels in the UIoT environment. There is an insulating material named dielectric utilized for optical channel propagation in UIoT. This mechanism is explained by the plasma frequency, operating as either a dielectric or conductor, following the frequency range. There are changes from a conductor to a dielectric at around 250 GHz in seawater. Attenuation and scattering are minor in the case of short-distance communication. Furthermore, the speed is up to 3 × 10^8^ m/s. Therefore, the optical signal is more reliable in short-range communication up to 10 m and suitable up to 100 m. Visible light communication (VLC) is the communication technology derived from an optical signal in UIoT. The ranges are from 450 nanometers to 550 nanometers at 500 Mbps and a distance of 100 m. Moreover, the speed is very high, up to 5 m. Therefore, VLC is very effective in short-range and one-to-one communication.

As stated, electromagnetic signals and optical signals have a limited transmission range. In addition, these signals are heavily affected by attenuation, scattering, and turbulence. This leads to a limit on the transmission distance. Therefore, acoustic communication technology is used for long-distance propagation in UIoT. The communication distance is up to 1 km at a speed of approximately 1500 m/second.

Magnetic Induction (MI) based communication technology is most commonly used in the underground of the seabed. It can cover a maximum of 10 m. The MI signal propagation speed is the same as the speed of light inside water, 3 × 10^8^ m/s. Moreover, the data rate is in the order of kilobits per second (kbps).

## 3. Q2: What Are the Challenges of the Current UIoT System

This section describes the UIoT system’s challenges, including channel characteristics, technical challenges, security challenges and privacy issues.

### 3.1. Channel Characteristics of UIoT

Delphin et al. pointed out that most of the characteristics of IoT systems are suitable for the UIoT environment since UIoT is the subclause of IoT [26]. Most of the available IoT protocols are designed and developed for stable nodes. Additionally, the performance of IoT networks can be reduced with the addition of new nodes and variations in terrestrial environment techniques. This statement highlights why the existing protocols and security models of terrestrial IoT should not be directly applied to UIoT.

#### 3.1.1. Underwater Channel 

Unlike terrestrial IoT, UIoT nodes typically communicate via acoustic, optical, RF and MI channels [27]. This results in long propagation delay, high battery consumption, high error rate, etc. Moreover, the behavior of each channel’s characteristics is different in the UIoT environment [2,3,4]. For example, the bandwidth of the acoustic channel is only a small percentage when compared to the RF channel [28]. Furthermore, due to the open characteristics of this UIoT environment, the attackers can easily inject the malicious node and steal the data or hack the communication channel [29].

#### 3.1.2. Energy Consumption and Storage 

UIoT nodes are designed with limited battery power, computational capacity and memory space [21]. Furthermore, the nodes consume more power for data gathering, processing and transferring. Compared to terrestrial networks, the nodes are rechargeable using solar energy. However, in UIoT networks, it is not easy to maintain or recharge due to the natural behavior of the environment. This may cause power constraints in UIoT networks.

#### 3.1.3. Environmental Condition

Due to internal waves, mammals activity and other objects’ behaviors lead to dynamic topology formation in UIoT networks [30]. The frequent changes of the UIoT network topology can cause rerouting, transmission loss and data accuracy issues [31]. Compared with the terrestrial IoT, in UIoT networks, the nodes are sparsely deployed for data gathering and transmission. Furthermore, since the UIoT nodes are mobile, localization, synchronization and secure communication are the other issues in UIoT networks.

### 3.2. Technical Challenges of UIoT

As a branch of the terrestrial internet of things (T-IoT), some particularities of UIoT are similar to T-IoT [32]. Unfortunately, due to the difference in the working environment, some unique particularities and constraints are outlined below.

#### 3.2.1. Limited Resources

In the UIoT environment, the battery and storage capacity of sensing devices are very limited.

*Limited battery:* The optical and acoustic communication channel in the UIoT environment consumes more power than RF communication. Furthermore, energy harvesting is impossible due to the unavailability of solar power creation in the UIoT environment. This causes data loss and reduces battery lifetime [33]. In addition, the existing low energy consumption or optimization methods used in the terrestrial environment, for example, the methods used in references [34,35], cannot be applied to UIoT networks.

*Limited storage capacity:* The memory size of devices in the UIoT environment is limited. Moreover, memory formatting is impossible in the UIoT environment. This causes failure in data gathering and data transmitting [2]. 

#### 3.2.2. Unreliable Channel Condition

In the UIoT Environment, the Cause of Unreliable Communication Channels Refer to the Factors that can Affect Data Transmission Loss Underwater.

*Limited bandwidth and transmission delay:* In an acoustic communication channel, the bandwidth is limited, such as from 100 kHz to 500 kHz, from 10 kHz to 100 kHz, and from 500 Hz to 10 kHz for short, medium and long-range communication in the UIoT environment, respectively. Furthermore, the data rate is a maximum of 100 kb/s. This causes a delay in data transmission [21]. 

*Attenuation and scattering:* Approximately ≤150 MHz and Hz to 10 kHZ can be used for long-range data transmission in an optical and acoustic communication channel. Even though light spreads much more compared to the sound signal in the UIoT environment, both signals suffer the problem of attenuation and scattering in long-range communication. This causes a transmission loss for long-range communication [36]. 

*High propagation delay:* In the UIoT environment, numerous factors such as turbidity, depth, pH level, density, temperature, etc., are the major causes of high propagation delay in optical and acoustic channel communication. This causes transmission loss or delay in transmission [37].

*Channel noise:* In the UIoT environment, channel noise refers to the noise factor that affects the underwater communication channel, such as environmental and ambient noise. Environmental noise is the noise generated by human beings such as shipping, fishing, naval activities, etc., and ambient noise is the background sound generated from an unknown source such as wind, underwater objects, sea animals, etc. [38] 

*Node mobility:* The UIoT environment consists of static and mobile nodes. The static nodes are placed in a fixed position and the mobile nodes move from one place to another for data collection. However, the characteristics of deep seawater such as internal wave, sediment formation and deliberate motion of other particles, force the nodes to move from one to another at any time in the UIoT environment. This term is also defined as external force mobility. Due to external force mobility, the connectivity can be easily broken, which causes data transmission errors [22].

#### 3.2.3. Insecure Environment

In the UIoT environment, security methods are particularly necessary to monitor naval applications. however, due to the environmental condition, it is difficult to monitor the uiot networks and devices. in this case, the attackers find it easy to access the nodes or devices in the uiot environment. such types of attacks are denial-of-service (dos) attacks, jamming attacks, flooding attacks, etc. this causes serious damage to the legitimate node in the uiot environment [39]. 

#### 3.2.4. High Cost

As shown in Figure 1 of Section 1, in the UIoT environment, the sensor nodes are devices that are sparsely deployed. Additionally, the products are from different vendors. Therefore, it is too costly to install, monitor and manage the network and devices in the UIoT environment [2,3,4].

#### 3.2.5. Dynamic Topology

**Figure 3:**
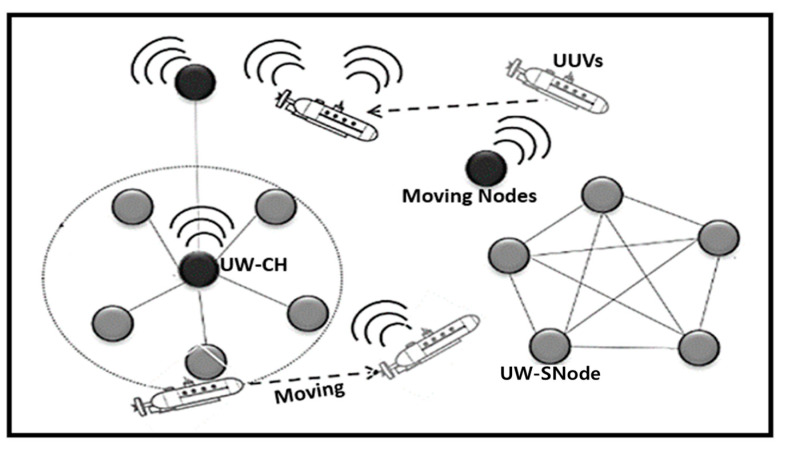
Dynamic topology formation.

Node mobility was discussed in Section 3.3.2. As shown in Figure 3, the UUVs and mobile nodes are automatically moving from one place to another or by external forces. Node mobility can form a new topology by modifying the existing topology. Therefore, node mobility is the major cause of dynamic topology formation in UIoT networks. This causes routing problems in the UIoT environment [40].

#### 3.2.6. Physical Damages

In the UIoT environment, the nodes are too deeply deployed in a harsh environment. Furthermore, nodes can be damaged easily because of marine objects such as deep-sea mammals, waste particles, internal waves, etc., which can cause severe damage to UIoT nodes, such as hardware failure, software error and broken links, making them dead nodes [3].

#### 3.2.7. Network Configuration

In the UIoT environment, since the nodes are mobile or stable, the connectivity can be easily broken or can generate a new topology, which can cause network configuration problems in UIoT networks [21]. 

### 3.3. Security Challenges of UIoT

This section describes the security challenges of UIoT that affect confidentiality, privacy, availability, resilience, authentication, safety, etc. The research shows a constant set of challenges for UIoT.

#### 3.3.1. Complex Environment

As discussed in Section 3.2.3, the UIoT is complex and insecure. For most of the applications, the sensor nodes are sparsely deployed and not well managed. This makes way for attackers to inject malicious nodes inside the UIoT networks. Furthermore, as discussed in Section 3.2.7, the underwater nodes can be physically broken due to the natural behavior of deep-sea and other living organisms. Therefore, monitoring and protecting nodes in a complex environment is an important discussion for the developers. 

#### 3.3.2. Data Privacy

In the UIoT environment, data privacy is extremely important since it can handle sensitive data in naval applications such as secret operations, identity sharing, enemy submarine tracking, etc. Since the UIoT environment is harsh, it is difficult to apply the privacy methods of terrestrial IoT environments such as k-anonymity, l-diversity, t-closeness and differential privacy to the UIoT environment. Therefore, the attackers can steal private data from UIoT devices. 

#### 3.3.3. Network and Device Management

**Figure 4:**
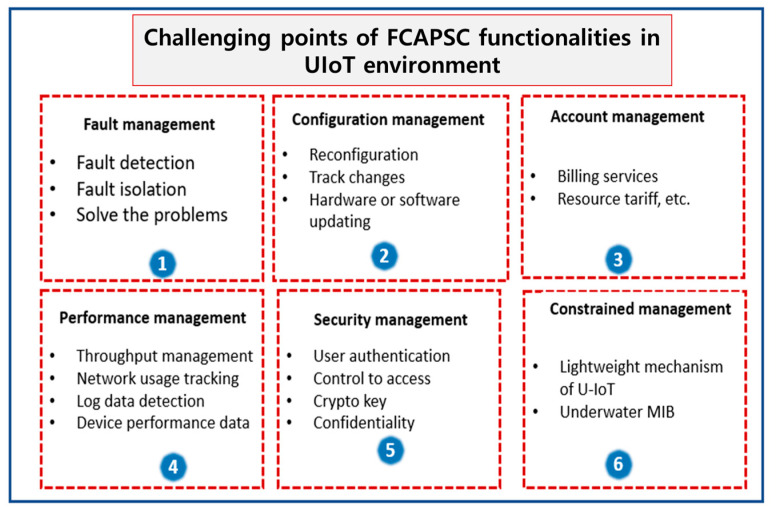
Challenges in adapting FCAPSC functionality.

The dynamic behavior of nodes and changes in topology as discussed earlier in Section 3.2.5 and other issues such as the limited battery, limited memory, routing, etc., can impact the management of networks and devices underwater. Therefore, as shown in Figure 4, it is difficult to manage the underwater network management system functionalities such as fault, configuration, accounting, performance, security and constrained (FCAPSC) management in the UIoT environment. Therefore, the attacker can target FCAPSC functionalities [21]. 

#### 3.3.4. Localization Techniques

In UIoT networks, node management is necessary to protect the nodes from physical damages and security attacks. In this case, it is necessary to adapt localization techniques to UIoT nodes to identify the location of each node underwater. However, due to heavy-weight and environmental limitations, the localization mechanism in terrestrial networks cannot be applied directly to the UIoT environment [41].

### 3.4. Security Goals, Attacks and Privacy of UIoT

**Figure 5:**
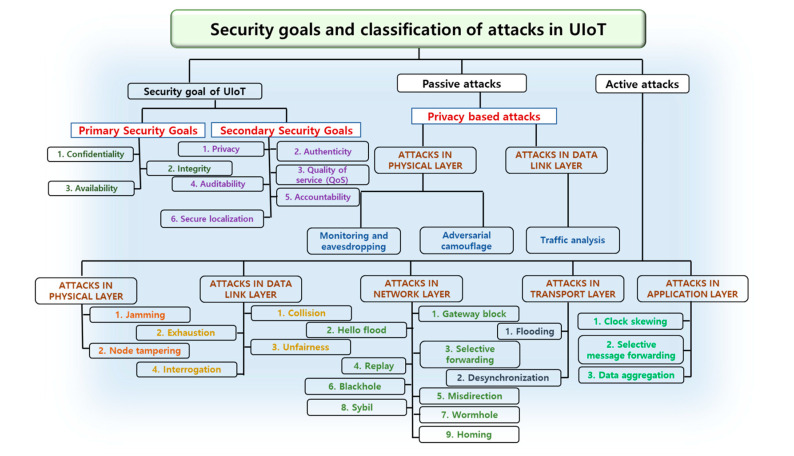
Goals and classification of security attacks in the UIoT environment.

This Section describes the security goals, attacks and privacy of UIoT networks. Figure 5 illustrates the security goals and classification of attacks in UIoT. 

#### 3.4.1. Security Goals of UIoT

It is classified into two parts (1) primary security goals and (2) secondary security goals [42,43,44]. Integrity, confidentiality and availability are the three primary security goals of UIoT, expected to be available in all UIoT applications. On the other hand, privacy, synchronization, authenticity, quality of service, auditability, accountability and secure localization are the secondary security goals of UIoT. The classification of UIoT security goals are described underneath.

##### Confidentiality

In UIoT networks, confidentiality is the essential feature for securing underwater data. A key sharing mechanism is a suitable approach that can be utilized to protect the data during transmission. In addition, for confidentiality, an auto-decision-making mechanism must be used for storing and retrieving data in the UIoT environment [42].

##### Integrity

In UIoT networks, data integrity is essential to maintain the accuracy and reliability of underwater data. Data integrity refers to the approaches to check whether the received data are altered during transmission via an underwater channel. For example, a message integrity check (MIC) can be used to verify the data integrity of received underwater data. In addition, an auto-integrity-checking mechanism such as logs integrity and software integrity can be used to verify the integrity of log reports and device software, respectively, in the UIoT environment [42]. 

##### Availability

In UIoT networks, data availability is necessary to provide the quality of services such as preventing UIoT devices from malicious attacks, securing harbor environment, securing diverse life at risk, etc. Self-healing, auto-recovery and centralized data sharing functions are necessary to support availability in UIoT networks [42].

##### Privacy

In UIoT networks, privacy refers to the information or service that a particular user or device can access. As discussed in Section 3.3.2, it is difficult to adapt the existing privacy approaches directly to UIoT networks. Hence, it is necessary to port a robust privacy approach for UIoT to protect the data from attackers. The types of privacy approaches that need to be considered in UIoT are categorized underneath:

*UIoT data privacy*: In UIoT networks, data privacy is necessary in naval applications to protect secret messages from attackers, e.g., enemy submarine attacking and secret message passing.

*UIoT device privacy*: In UIoT networks, a device identity is generally used to track and transfer information to UIoT devices. This identity is traceable; therefore, it is easy for the attackers to steal the information. In this case, a robust identity protection approach is necessary to hide the device identity from malicious nodes.

*UIoT location privacy*: In UIoT networks, location information is necessary to track the mobility of UIoT devices. The location information is open and is essential for data transmission between the nodes in the underwater environment. In addition, hiding the location of nodes based on necessity is a challenging task. Hence, it is necessary to port a privacy-based location sharing mechanism for UIoT devices.

##### Authenticity

In UIoT networks, authentication refers to the verification between sender and receiver node. As discussed in Section 3.3.1, the environmental condition is complex. In addition, it is difficult to adapt the terrestrial authentication scheme to the UIoT environment. Therefore, the attacker finds it easier to block the channel. Hence, it is necessary to design a lightweight authentication scheme for UIoT networks.

##### Auditability

In UIoT networks, it is necessary to analyze security functions’ security activities and performance to provide high-quality services. Hence, an auto-auditing or self-auditing mechanism can be considered to evaluate the security systems in the UIoT environment.

##### Others

In UIoT networks, other security goals such as audibility, data freshness, self-organization, time synchronization, secure localization, etc., can be considered to provide the quality of services (QoS) in the UIoT environment.

#### 3.4.2. Passive Attacks

The unauthorized attacker attacks the UIoT channel without altering the data. These attacks have silent carriers because they do not carry any signals. The attacker is hidden during a passive attack and can cause node tampering, jamming, message distortion and replaying. Furthermore, the attacker can anticipate the idea of UIoT networks by identifying packet traffic, observing packet exchange nodes and predicting the location of nodes. Passive attacks are also known as privacy-based attacks. The types of passive attacks are mentioned below:

*Monitoring and eavesdropping*: It is the most commonly used attack against data privacy in UIoT environment. When the network traffic is at its peak, the attacker can steal important information by tapping the network configuration. This type of attack is categorized under privacy-based attacks.

*Adversary and camouflage*: In this case, the invisible attacker injects an adversary node into the UIoT network. In effect, the adversary node can track and modify the information in UIoT networks, such as stealing packets, rerouting packets and altering nodes.

*Traffic analysis*: In these attacks, the attacker infuses the UIoT networks by accessing the pattern in the communication channel. Through this, the attacker can listen to the location of each node, the routing path, the behavior, etc.

#### 3.4.3. Active Attacks

The unauthorized attacker can alter, infuse, erase or destroy information in UIoT networks. The active attack can delete or modify the data during transmission and after transmission. Active attacks in UIoT are categorised into five categories: (1) Denial-of-service, (2) Message distortion, (3) Node tampering, (4) Message replay and (5) Masquerade attacks. The types of active attacks are classified under each layer of UIoT networks, such as a physical layer, data link layer, network layer, transport layer and application layer.

**Figure 6:**
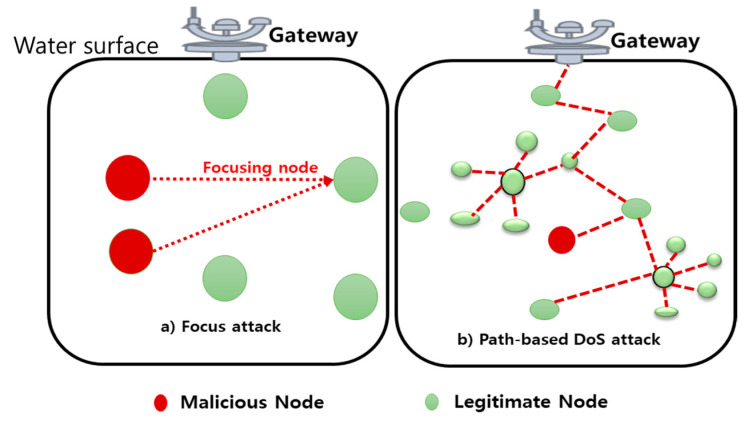
Types of DoS attacks in the UIoT environment.

Denial of service attacks is one of the deadliest active attacks and can cause a ton of damage. DoS attacks can be used at any layer of UIoT networks. DoS is an active attack that attempts to make assets out of reach to the authentic node. The attacker tries to block the authentic nodes from retrieving the services offered by the network [45]. Figure 6 shows the types of DoS attacks in UIoT.

Node tampering: The UIoT nodes consist of hardware components such as a controller, battery, transmitter and receiver. In node tampering, the attacker can track and modify the software code of underwater nodes. Due to this, the software and hardware parts can be broken, which causes severe damage to the nodes in the UIoT environment. In effect, it causes network lifetime damages and data loss.

*Message distortion*: In these attacks, the attacker can alter the data sent by one UIoT node to another. It can cause severe damages in case of emergency UIoT applications, e.g., message distortion in the naval application can break the security system. This could cause confusion by passing wrong information to the end-users.

*Message Replay*: In these attacks, the attacker acts like the source node to send the same information already sent by the source node, or the attacker purposely delays transferring data by hacking. A message replay attack is also known as a play-back attack.

*Masquerade*: In these attacks, the attacker uses the fake identity to steal the information from a legitimate node. A masquerade attack is a kind of privacy attack.

**Figure 7:**
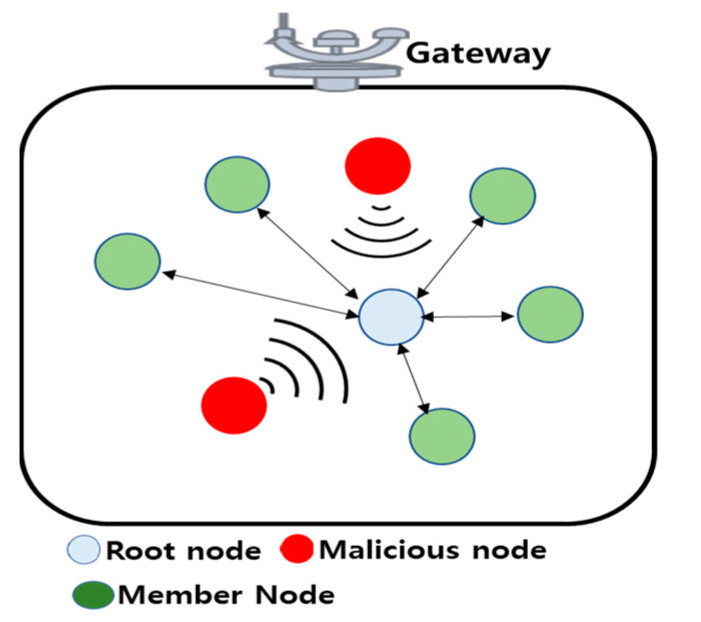
Jamming attack in the UIoT environment.

*Jamming attack:* In these attacks, the malicious nodes frequently send the noise signal to disturb legitimate nodes in UIoT networks. Additionally, this attack can hack few special nodes inside the UIoT networks, such as root node, gateway, underwater cluster head, etc., which causes jamming in UIoT networks. In effect, it stops data transmission and gathering. Figure 7 shows the jamming attack where a malicious node continuously attacks the root node, disrupting the communication with the member node.

Collision attack: This attack happens in the data-link layer of UIoT networks. A collision happens when two underwater nodes send packets at the same time. Hence, to avoid the collision in UIoT networks, the underwater nodes follow the data transmission rules, namely, that underwater nodes should not use the same time for data transmission. However, in a collision attack, the attacker will violate the rules and send the packets simultaneously. In effect, the UIoT networks need frequent retransmission and cause power loss.

**Figure 8:**
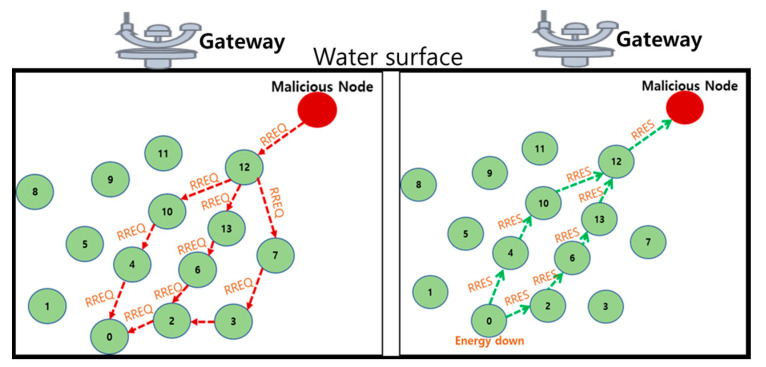
Battery-oriented attack.

Exhaustion attack/battery-oriented attack: This attack aims to drain the total energy of underwater nodes in UIoT networks. For example, Figure 8 shows the battery-oriented attack of UIoT networks. Here, the malicious node sent a routing request (RREQ) message to node 0. In response, node 0 sent the routing response (RRES) message to the malicious node. Finally, the malicious node will continuously send the corrupted packets until node 0 becomes dead. In effect, it reduces network lifetime.

*Node compromise attack*: An attacker can capture, break and compromise UIoT nodes to read or change information from memory. Moreover, what is terrible, is that the compromised nodes can penetrate into the network as authentic nodes to screen or disrupt it, which can prompt considerably more prominent harm. An attacker can find the network by checking the power of the acoustic signal and capturing them. More regrettable, is that xfwithout a trace of hack-confirmation equipment or other security systems, the attacker can undoubtedly break and compromise them to inspect private information (e.g., the secret key, the encryption algorithm, the trust esteem) and alter this information in the inward memory. Additionally, the compromised node can be penetrated into the network as an actual node to screen it or perform persistent attacks.

**Figure 9:**
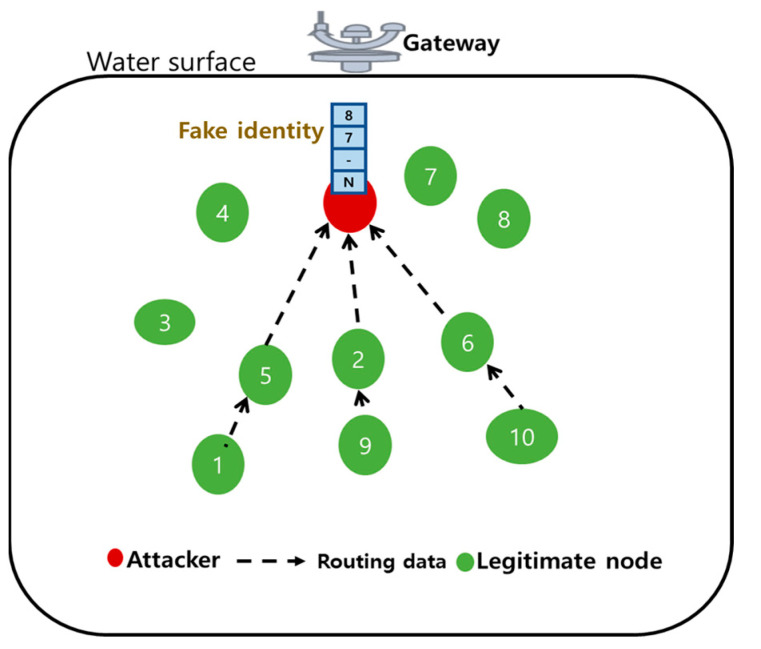
Sybil attack in UIoT environment.

*Sybil attack*: The Sybil attack is a type of routing attack. In this case, the attacker uses a fake identity to steal the information while routing. Figure 9 shows that the attacker can locate any place in UIoT networks and use multiple identities to mislead routing. In effect, it causes packet loss or transmission delay [46,47,48].

**Figure 10:**
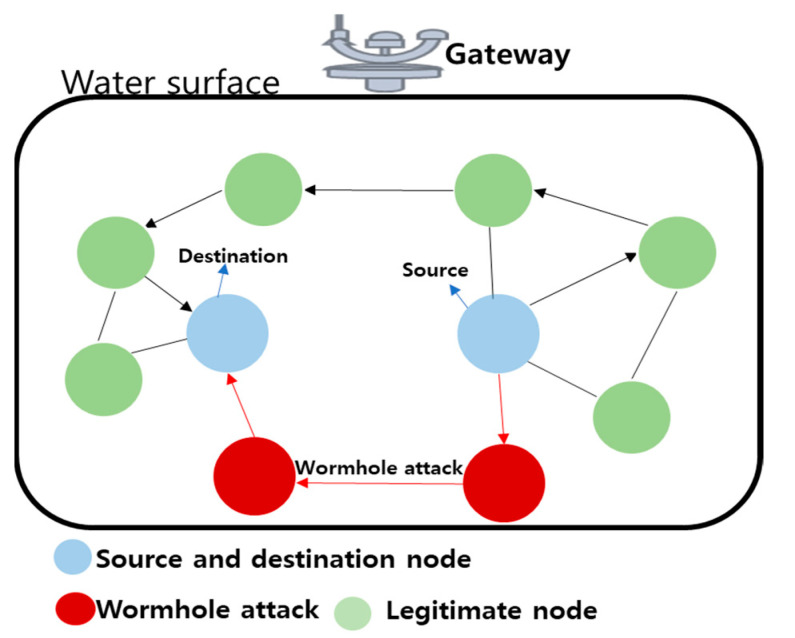
Wormhole attack in the UIoT environment.

Wormhole attack: An attacker uses two malicious nodes to tunnel traffic through the UIoT networks in a wormhole attack [49,50,51,52]. The two plotting nodes capture packets at one end and block them at another end. Wormhole attacks can make fake neighbor associations and give the probability of an alternate path for routing. Figure 10 explains how a wormhole attack occurs, causing a breach in the communication link, only because it looks like the distance of the wormhole node is shorter than legitimate nodes.

Unfairness: This is a type of DoS attack. The attacker aims to reduce the performance of the legitimate nodes instead of completely blocking them from data transmission. In effect, it can create transmission delay in UIoT networks.

**Figure 11:**
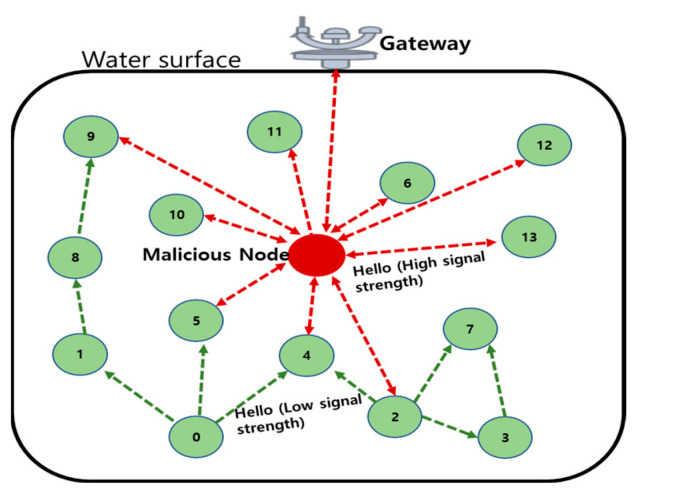
Hello flooding attack in the UIoT environment.

*Hello flooding attack*: In a UIoT environment, every node will send HELLO packets to identify its neighbor node. In a hello flooding attack, the adversary node in a UIoT network will send numerous HELLO packets to legitimate nodes to exhaust their battery power. In this case, the adversary node will convince the legitimate node by transmitting the signal with high intensity. Therefore, the legitimate node will assume the adversary node as the neighbor node and transmit data. In effect, it causes power failure and reduces the network lifetime. Figure 11 shows that the malicious node sends HELLO packets with high signal strength to attract the legitimate nodes in UIoT networks [53].

**Figure 12:**
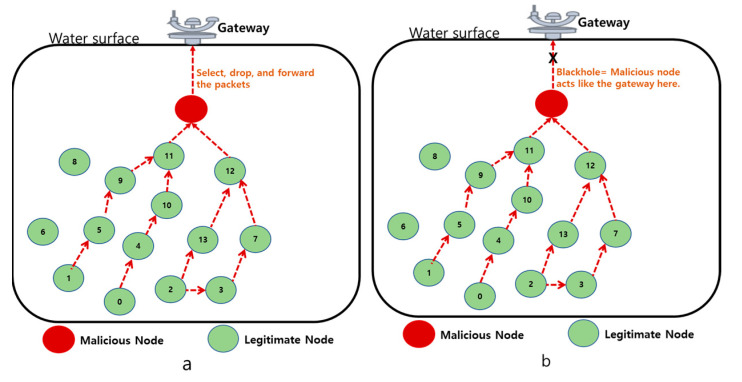
(**a**) Selective forwarding attack, (**b**) black hole attack in the UIoT environment.

*Selective forwarding*: In these attacks, the malicious node is located nearby the gateway of UIoT networks. When some packets are detected, the legitimate nodes will find a new route for transmitting the data to the gateway. As shown in Figure 12a, the malicious node can selectively drop some packets before reaching the destination in this attack. In effect, it causes packet loss in UIoT networks.

*Blackhole attack*: In these attacks, the malicious node acts as the cluster head or gateway to drop the packets while routing. Figure 12b shows that the malicious node can blackhole by modifying or dropping the packets routed from legitimate nodes. The dropped packets are referred to as black hole attacks in UIoT networks.

*Gateway block attack*: In this attack, the malicious node is located near the gateway and blocks all the data transferred from legitimate nodes to the gateway. In this case, the attacker manages to steal all the routing information sent to the gateway as the destination. In effect, it causes complete packet loss. Therefore, a gateway block attack is referred to as the main threat in UIoT networks.

*Misdirection attack*: In this attack, the malicious node can be located anywhere in the UIoT network and track the routing path to change the route to the malicious node. In effect, this attack causes packet loss or data transmission delay.

*Homing attack*: The malicious node observes the traffic in UIoT networks and attacks the most special nodes in UIoT networks, such as cluster head and gateway. Additionally, this attacker can jam or destroy those special nodes using a DoS attack.

*Desynchronization attack*: This attack disturbs the active connections between the nodes in UIoT networks by sending fake packets. In this case, the fake packets will carry fake sequence numbers to distract the synchronization process between the underwater nodes. In effect, it affects the accuracy in UIoT networks.

Clock skewing attack: In these attacks, the attacker tries to obtain the timestamp information of a legitimate node. Therefore, the time stamp information can be changed in a legitimate node. In effect, it causes a time synchronization problem in UIoT networks.

Data aggregation attack: In these attacks, the attacker tries to aggregate the legitimate node’s privacy-based information in UIoT networks. The attacker can steal information such as username, passwords, etc.

## 4. Q3: What Are the Methodologies Used to Overcome the Challenges in UIoT?

Several methods are proposed to solve the technical and security challenges of UIoT. Some of them provide a general idea, and others give a solution for existing problems. Some of the existing techniques to overcome the UIoT challenges are discussed below.

### 4.1. Methods to Overcome the Technical Challenges of UIoT

#### 4.1.1. Low Battery Consumption Methods

In [54,55,56,57,58,59,60,61,62,63,64], the existing techniques for solving the battery problem in UIoT are discussed, and some methods are indicated herewith. In [56], Pendergast et al. proposed a powerful and rechargeable module using Panasonic (CGR18650E) to provide sufficient energy, and the experiment result shows that it is reliable and safe in the underwater environment. In [58], Raffaele Guida et al. designed a battery-less underwater node that can recharge via an acoustic signal from a short or long distance. In [59], Guanglin Xing proposed a named data networking (NDN) approach for relay network topology in underwater acoustic sensor networks to identify the node’s power consumption in a shallow sea and deep-sea environment. Finally, in [60], Ahmed G, a two-level Redundant Transmission Control (RTC) was proposed to control the communication in underwater acoustic sensor networks, and the performance result shows that energy consumption is lower for the RTC approach.

#### 4.1.2. Memory Management Methods

In [7,65,66,67,68], the existing techniques for solving the storage management in UIoT are discussed, and some methods are indicated herewith. In [7], I.F. Akyildiz et al. suggested that underwater sensors need to perform some data caching due to the intermittent underwater channel characteristics. In [65], Zahoor Ali Khan et al. researched Q-learning (QL), comprising of reactive and proactive strategies to reduce the network overhead related to network lifetime. In [66,67] memory management, an essential function to store and retrieve information through smart sensing underwater devices, was studied to solve the challenges of the underwater network management system (U-NMS).

#### 4.1.3. Unreliable Data Transmission Methods

In [68,69,70], the existing techniques for solving the unreliable data communication in UIoT are discussed, and some methods are indicated herewith. In [68], Li, N et al. show that unreliable channels cause propagation delays. Therefore, three aspects of solving this problem suggested reducing unnecessary routing detection, routing distance between relay nodes and retransmission. In [69], S. Jiang recognized the need for an optimal design to provide reliable end-to-end transmission. Thus, a reliable transmission control was systematically reviewed, focusing on the data link, network and transport layers. Finally, in [70], Fattah S et al. discussed the impact of noise from underwater environments on reliable data transmission, and based on this, link reliability was an essential consideration for data transmission to achieve the rate of high transmission in real-time scenarios.

#### 4.1.4. Noise Modeling Methods

In [71,72,73,74,75,76,77,78,79,80,81,82], the existing techniques for solving the environmental noise and ambient noise modeling in UIoT are discussed, and some methods are indicated herewith. In [72], Chao Wang et al. designed a PG mixed noise model based on a single-photon avalanche diode (SPAD) in an underwater visible light communication system by considering the attenuation and turbulence effect. Here, an algorithm for the noise model was also presented. In [76], Bagocius D et al. presented an underwater noise model to identify the noise level of shallow water during different seasons. Finally, in [78], Pennucci et al. provide the conceptual design and describe the effect of using ships in the UIoT environment by providing various shipping noises for noise modeling underwater.

#### 4.1.5. Localization Methods

In [83,84,85,86], the existing techniques for solving the localization problem in UIoT are discussed, and some methods are indicated herewith. In [83], T. Islam et al. anticipated that localization is a crucial element in the protocol design given the proposed geographic routing protocols for underwater sensor networks. Suggestively, they resulted in accuracy and coverage of localization as essential factors for performance based on the surveyed centralized and distributed localization algorithms. In addition, P. Liu, B et al. proposed the integrated navigation of the Inertial Navigation System (INS) in AUV with limited doppler velocity log (DVL) to update the depth of the system based on the pressure sensor integrated with AUV [84].

#### 4.1.6. Low-Cost Communication Methods

In [17,87,88,89,90,91,92,93,94,95,96,97,98,99,100,101], the existing techniques for solving the high-cost issues in UIoT are discussed, and some methods are indicated herewith. In [89], Bridget Benson et al. designed a low-cost acoustic modem to reduce underwater acoustic sensor network cost and power consumption. In [99], Waseem et al. designed a low-cost application to monitor water quality using underwater wireless communication. In [100], Brian R et al. designed and developed a low-cost glider to perform in shallow water, around 3-m depth, 3-m radius and a minimum of 60 h durability. Finally, in [101], Abdillah designed and developed a low-cost coral reef monitoring application for shallow water.

#### 4.1.7. Device Management and Physical Damage Protection Methods

In [102,103], the existing techniques for solving the device management issues in UIoT are discussed, and some methods are indicated herewith. In the case of device management, in ISO/IEC 30140-1, fouling cleaners and housing cases shall be used for cleaning marine wild animals attached to underwater devices, waterproofing and construction of underwater sensor nodes resistant to high water pressures, respectively. In addition, as a functional requirement for underwater device management, identification of available resources and status of the devices are suggested in ISO/IEC 30142. In [26], K. M, D.R. et al. designed and developed the underwater network management system (U-NMS). The proposed system enables automatic software updates and monitoring of underwater devices using fault, configuration, accounting, performance, security and constrained management (FCAPSC) functions of U-NMS for physical damage protection.

#### 4.1.8. Connection and Reconfiguration Methods

In [104,105,106], the existing techniques for solving the connectivity issues in UIoT are discussed, and some methods are indicated herewith. In [105], L. Furno, a self-reconfiguration algorithm is formulated for underwater robots based on energy heuristics. In [106], a full-duplex, parameter configurable, multiple-user modem is developed and tested to improve the throughput level in the UIoT environment.

### 4.2. Methods to Overcome the Security Challenges in UIoT

#### 4.2.1. Methods to Prevent DoS Attacks

The existing techniques to prevent DoS attacks in UIoT are discussed herewith. In [107], Martin et al. proposed a cautious calculation that checks the potential DoS attack. This approach breaks down centered and broadcasted DoS attacks to initially distinguish the attack and create pushback alerts or choke the malicious nodes as they enter the UIoT networks. Data entropy is a proportion of the vulnerability related to an irregular variable. It tends to be deciphered as the normal most limited message length in bits that can send an irregular variable to a recipient [108]. Entropy can be determined by figuring a progression of constant bundles. The entropy esteem gives a depiction of the comparing arbitrary appropriation of these sources IP addresses. The bigger the entropy, the more irregular the source IP. The more modest the entropy, the smaller the dispersion scope of the source IP locations of the parcels, and a few locations have a genuinely high likelihood of an event. The expression for calculating the entropy is shown below:

$$E=-\overset{Tn}{\underset{k=1}{\sum }}=p_{k}\;log_{2}\;p_{k}$$

Here *p_k_* is the possible outcome probability, *Tn* is the number of packets analyzed, and *E* is the entropy.

#### 4.2.2. Methods to Prevent Jamming Attacks

The existing techniques to prevent jamming attacks in UIoT are discussed herewith. In [109], Misra et al. present a shortcoming identification calculation where nodes deliberately trade revelation and affirmative packets. In [110], Bagali et al. present a productive channel task conspire, an original cross-layer plan for helpful correspondence for jamming detection. Finally, in [111], Xiao et al. proposed utilizing the game-hypothetical investigation of sticking to UIoT and proposed a machine learning-based energy management mechanism to adapt to jamming attacks in UIoT networks. The associations between a UIoT and a responsive jamming device are defined as two jamming games.

Exponentially Weighted Moving Average (EWMA) was proposed by Osanaiye et al. [112] as a measurable productive procedure for identifying little changes in time series information. It works by first characterizing an edge that portrays standard conduct before intermittently refreshing the normal of the noticed traffic. The EWMA algorithm can be the countermeasure for jamming attacks. The below expression shows how the EWMA is calculated:

x(d)=λ.y(d) + (1−λ). x(d−1) d=1,2,3,…Nλ

*x*(*d*) is the data with moving average time *d*, *λ* is the parameter value between 0 and 1, *y(d)* denotes the signal *y* at a time ‘*d*’, *N* is the number of observations in EWMA.

#### 4.2.3. Methods to Prevent Node Compromise Attacks

To defend against node compromise attacks in UIoT networks, a mechanism such as a high-level hardware protection scheme, trustworthiness, data management and configuration management should be adapted for UIoT networks.

#### 4.2.4. Methods to Prevent Sybil Attacks

Message authentication and proper localization mechanisms are necessary to prevent the Sybil attack in the UIoT environment. The existing Sybil attack prevention methods applicable for UIoT networks are explained herewith. In [46], Demirbas et al. proposed the received signal strength indicator (RSSI) based light-weight approach to detect the Sybil attack; this approach can be applicable in UIoT networks. In [47], W. Du et al. proposed a pairwise random key predistribution scheme to secure the communication link that can be used for UIoT networks. Resource-based testing is one of the solutions for Sybil attack prevention in UIoT networks. In [48], Newsome et al. provide an example of resource-based testing. This method can be used in UIoT.

#### 4.2.5. Methods to Prevent Wormhole Attacks

The existing techniques to prevent wormhole attacks in UIoT are discussed herewith. In [49], Gorlatova et al. used the HELLO message based on packet timing analysis to control the wormhole attack, which can be used in UIoT networks. In [50], Kong et al. proposed a two-tire-based localization method to identify the wormhole attack in a short time in UIoT networks. In [51], Shang-Ming Jen et al. proposed a hop-count-based analysis method to prevent the wormhole attack, which can be applicable in UIoT networks. Finally, in [52], Wang et al. proposed a distributed method to identify the wormhole attack in UIoT networks.

#### 4.2.6. Methods to Prevent Flooding Attacks

The existing techniques to prevent flooding attacks in UIoT are discussed herewith. Bidirectional authentication is necessary to protect the nodes from flooding attacks in UIoT networks. In [53], Prabhjot Kaur et al. proposed a centralized scheme to protect the hello flooding attack that can be used in UIoT networks. In [113], Coutinho et al. proposed a GEDAR, a geographical routing approach that prevents flooding attacks underwater. In the GEDAR approach, the communication is established based on the location information of UIoT nodes.

#### 4.2.7. Methods to Prevent Black-Hole Attacks

The existing techniques to prevent black-hole attacks that can be considered for UIoT are discussed herewith. In [114], a dynamic learning system (DPRAODV) was proposed against black-hole attacks in mobile ad hoc networks. In [115], L. Tamilselvan et al. proposed the cooperative black-hole prevention method using a fidelity table in mobile ad hoc networks. In [116], Hanane Kalkha et al. proposed the tyenHidden Markov Model technique to identify the black-hole attacks in wireless sensor networks.

## 5. Q4: What Are the Findings Based on the Existing Research Works?

**Figure 13:**
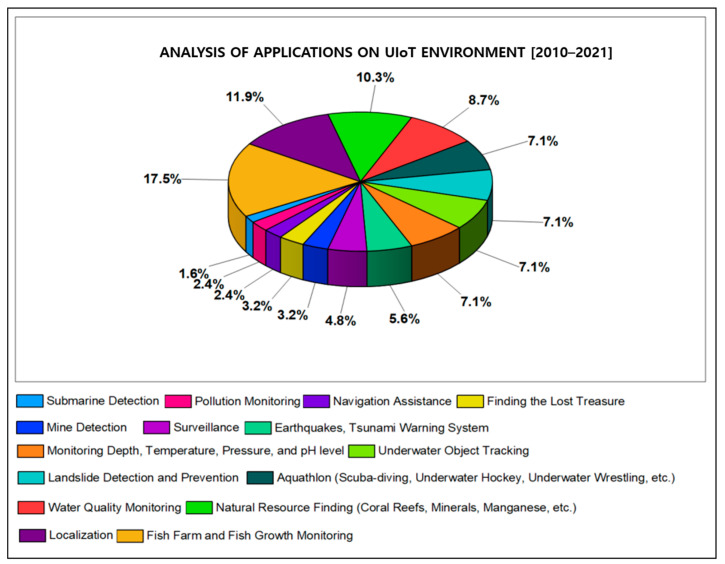
Results based on the systematic analysis of UIoT applications.

**Figure 14:**
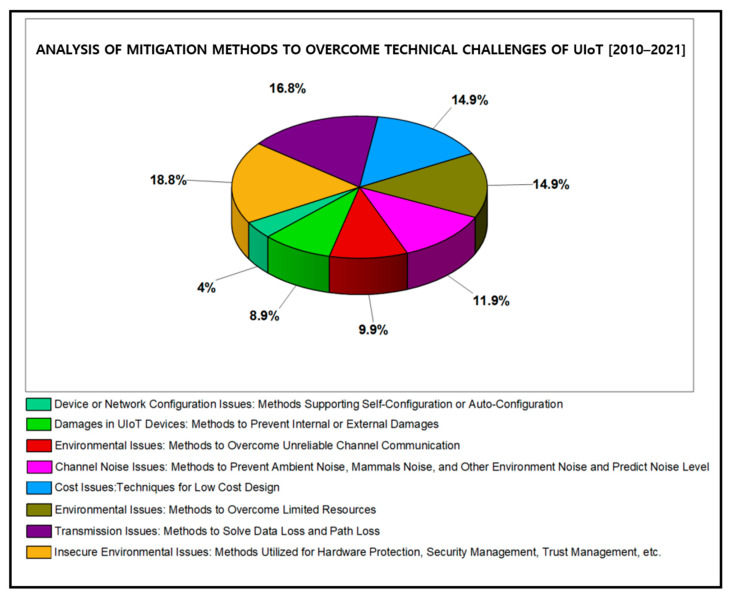
Results based on the systematic analysis of mitigation methods to overcome the technical challenges in UIoT.

**Figure 15:**
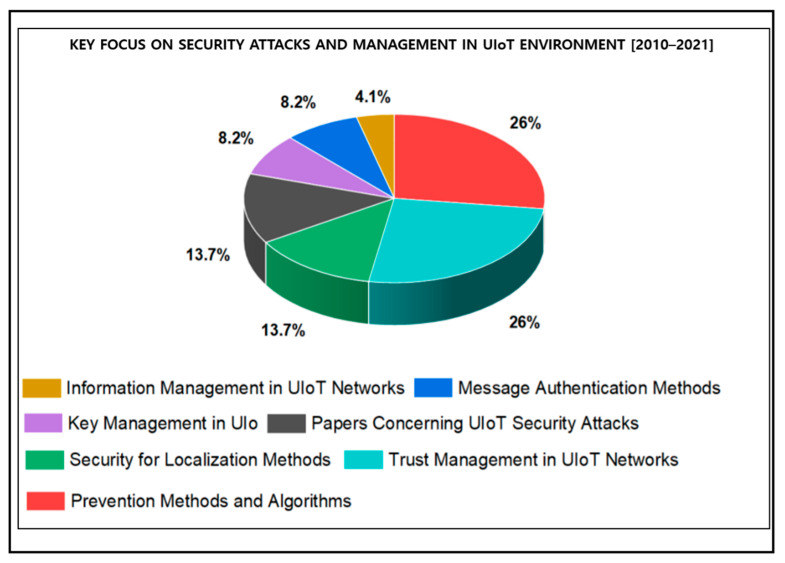
Results based on the systematic analysis of security attacks and management.

This section highlights the significant findings of this research by reviewing the papers concerning recent trends, technical challenges, privacy and security issues of UIoT. The analysis is provided in Table 3, Table 4 and Table 5 based on the years from 2010 to 2021, and the results are displayed in Figure 13, Figure 14 and Figure 15.

## 6. Q5: Future Direction

According to the results obtained from the current research study conducted based on queries in Table 1, the suggestion for the future direction of UIoT is discussed in the Section s beneath:

### 6.1. Build Hybrid Communication Models for Future UIoT

Based on the research study in Section 2, acoustic, optical, RF and MI are the communication technologies used in the UIoT environment. As shown in Table 2, each medium has its advantages and disadvantages. To overcome the technical challenges discussed in Section 3.2, it is necessary to port multi-medium (hybrid) communication technology in UIoT [21]. The multi-medium communication technology can improve the transmission speed, increase the battery life, and deliver reliable data transmission in UIoT.

### 6.2. Build Underwater Automatic Battery Recharging Module for Future UIoT

Based on the research study in Section 3, the devices or nodes in the UIoT environment have limited resources. Additionally, it is difficult to recharge in a constrained underwater environment. In effect, it reduces battery life and network lifetime if any one of the nodes is dead. In [267], Yongil Kim et al. introduced a metal-free sodium-seawater battery (Na-SWB). In [268], J Cho et al. proposed a battery degradation prediction and power optimization mechanism for surface buoys based on sea batteries. In [269], Moon Son et al. proposed a rechargeable seawater battery (SWB) mechanism that produces energy from seawater. Finally, in [338], the Miresearch group developed battery-free sensor nodes for underwater exploration. Therefore, to solve the battery issues in UIoT, it is necessary to build an undersea battery or an automatic recharging mechanism or deploy battery-free nodes.

### 6.3. Build Standard Security Models for Future UIoT

Section 3.3 and Section 3.4 describes the security issues and possible security attacks in UIoT networks. This research study shows that it is necessary to build a robust security model that includes high-level security architecture, confidentiality, integrity, availability, quality of service (QoS), etc., to protect the UIoT nodes from attacks such as DoS attacks, routing, jamming attacks and so on.

### 6.4. Build Privacy Models for Future UIoT

Based on the discussion in Section 3.3.2, it is necessary to handle privacy issues in essential applications of UIoT such as diver networks, naval applications, tracking applications, etc. However, since the terrestrial privacy models are heavyweight, it is difficult to apply in UIoT environments. Moreover, as discussed in Section 3.4.1, it is necessary to consider data privacy, device privacy and location privacy in UIoT. Hence, it is necessary to build lightweight privacy models for UIoT systems by adapting privacy models in terrestrial networks such as k-anonymity, l-diversity, t-closeness and differential privacy.

## 7. Conclusions

This paper reviews existing research papers based on recent trends, applications, challenges, security and privacy issues of UIoT. Additionally, the possible solutions to overcome the technical challenges, privacy and security issues are discussed based on the systematic studies. The research goals are developed in Table 1, including four research queries from Q1 to Q4, and the solutions are provided under Section 2, Section 3, Section 4 and Section 5. Section 2 provides the survey based on the latest articles, the recently developed applications and the existing communication technologies of UIoT. Section 3 describes the existing challenges of UIoT systems, including technical challenges, privacy and security attacks in UIoT networks. Section 4 provides the methodology to overcome the challenges described in Section 3. In Section 4, the significant findings are highlighted by reviewing the total number of papers concerning UIoT applications, technical challenges, privacy and security issues of UIoT. Finally, the future direction in Section 5 shows that the hybrid communication technologies in UIoT that include acoustic, optical, IR and MI medium can overcome the technical challenges of the UIoT system. Therefore, further research needs hybrid modem technology to support fast, reliable and low power consumption-based communication in UIoT. Moreover, in the future, the privacy and security issues can be solved by building standard security models and security architecture for UIoT. Furthermore, it is necessary to build battery-free sensors or undersea energy models for energy storage and automatic recharging in the future.

## Figures and Tables

**Table 1:** Research Goals.

Queries (Qs)	Discussion
**Q1**: What are the current trends of the UIoT system?	UIoT is the growing trend in the current IoT system. Recently, numerous UIoT applications have been developed for the industries. Therefore, Q1 provides the survey based on the latest article and the recently developed UIoT applications. Furthermore, the communication technologies of UIoT are discussed, which includes the pros and cons of UIoT channels such as RF, acoustic, optical and MI.
**Q2**: What are the challenges of the current UIoT system?	Challenges include technical challenges, security attacks and privacy issues. Therefore, Q2 discusses the technical challenges based on UIoT channel characteristics and the possible security challenges and privacy issues in UIoT.
**Q3:** What are the possible methods to overcome the challenges, security attacks and privacy issues in the UIoT system?	In the UIoT system, most of the challenges and security issues are still of concern. Likewise, privacy methodologies are not yet considered for the current UIoT system. Therefore, Q3 highlights the countermeasures taken to overcome the challenges, security attacks and privacy issues of the current UIoT system.
**Q4 and Q5:** What are the findings and future directions?	Q4 discusses the findings based on the systematic review and Q5 highlights the future direction of this paper.

**Table 2:** Communication technologies of UIoT [2,3,4,5,6,7,8,9,10,11,12,13,14,15,16,17,18,19,20,21,22,23,24,25].

Attributes	Acoustic	RF	Optical	MI
Channel speed	≈1500 m/s	≈3.33 × 10^8^ m/s	≈3.33 × 10^8^ m/s	≈3.33 × 10^8^ m/s
Communication range	≈kilometer (km)	≈10 m	≈10–100 m	≈10–100 m
Data rate	≈kbps	≈Mbps	≈Gbps	≈Mbps
Signal operation	Audible	Non-visible and non-audible	Visible	Non-visible and non-audible
Frequency band	10−15 kHz	30−300 Hz	≈5 × 10^14^ Hz	-
Size of the Antena	≈0.1 s	≈0.5 s	≈0.1 s	≈0.1 s
Channel characteristics dependency	Undersea noise, temperature, pressure, Doppler spread, salinity, etc.	Conductivity	Undersea noise, attenuation, turbidity, scattering, etc.	Conductivity
Bandwidth	≈1–100 Kilohertz (kHz)	≈Megahertz (MHz)	≤150 Megahertz (MHz)	≈Megahertz (MHz)
Purpose of each channel	Long-range communication	Surface water communication	Short-range communication	Underground communication in deep sea
Transmission power	>10 watts (W)	megawatts (MW)−watts(W)	megawatts (MW)−watts(W)	10^−8^ watts (W)
Power loss dependency	≈0.1 dB per meter (m) or per hertz (Hz)	≈28 dB per kilometer (km) or one million hertz (HZ)	Depending on the turbulence of water	Depending on the permeability of undersea soils

**Table 3:** Systematic analysis on UIoT applications.

Main Clause	Subclause	Paper Count	References Number
Environmental monitoring	Pollution monitoring	3	[117,118,119]
	Water quality monitoring	11	[120,121,122,123,124,125,126,127,128,129,130]
	Monitoring depth, temperature, pressure, and pH level.	9	[131,132,133,134,135,136,137,138,139]
	Fish farm and fish growth monitoring	22	[140,141,142,143,144,145,146,147,148,149,150,151,152,153,154,155,156,157,158,159,160,161]
Resource exploration	Finding the lost treasure	4	[162,163,164,165]
	Underwater object tracking	9	[166,167,168,169,170,171,172,173,174]
	Natural resource finding (Coral reefs, minerals, manganese, etc.)	13	[175,176,177,178,179,180,181,182,183,184,185,186]
Disaster prevention	Earthquakes, Tsunami warning system	7	[187,188,189,190,191,192,193]
	Landslide detection and prevention	9	[194,195,196,197,198,199,200,201,202]
Naval applications	Submarine detection	2	[203,204]
	Mine detection	4	[205,206,207,208]
	Surveillance	3	[209,210,211]
Others	Aquathlon (Scuba-diving, underwater hockey, underwater wrestling, etc.)	6	[212,213,214,215,216,217]
	Navigation assistance	9	[218,219,220,221,222,223,224,225,226]
	Localization	15	[85,86,227,228,229,230,231,232,233,234,235,236,237,238,239]

**Table 4:** Systematic analysis of the technical challenges in UIoT networks.

Problems	Solutions and Effective Methods	Paper Count	References Number
Transmission issues	Methods to preventing path loss and data loss in UIoT networks.	17	[240,241,242,243,244,245,246,247,248,249,250,251,252,253,254,255,256]
Environmental issues	Methods to solve unreliable channel conditions in UIoT networks.	10	[257,258,259,260,261,262,263,264,265,266]
	Methods to solve limited resources in UIoT networks.	15	[26,54,55,56,57,58,59,60,61,62,63,64,267,268,269]
Insecure environment issues	Methods used to support trust management, security management, hardware protection, etc., in UIoT networks.	19	[42,107,113,270,271,272,273,274,275,276,277,278,279,280,281,282,283,284,285]
Cost issues	Lost cost design approaches for UIoT networks	15	[87,88,89,90,91,92,93,94,95,96,97,98,99,100,101]
Channel noise issues	Methods to prevent ambient noise, mammals noise, other environmental noise in UIoT networks. Methods to predict noise level in UIoT networks.	12	[71,72,73,74,75,76,77,78,79,80,81,82]
Damages in UIoT devices	Methods to prevent internal or external damages of UIoT devices.	9	[26,286,287,288,289,290,291,292]
Device or network configuration issues	Methods supporting self-configuration or auto-configuration mechanism for devices in UIoT networks.	4	[26,104,105,106]

**Table 5:** Systematic analysis of security issues and management in UIoT networks.

Main Clause	Subclause	Paper Count	References Number
Key focus on security attacks and management	Papers discussing privacy and security attacks on UIoT networks.	10	[271,293,294,295,296,297,298,299,300,301]
	Papers discussing attack prevention methods and management in UIoT networks.	19	[42,107,113,270,271,272,273,274,275,276,277,278,279,280,281,282,283,284,285]
	Papers discussing message authentication techniques in UIoT networks.	6	[42,302,303,304,305,306]
	Papers discussing localization security in UIoT networks.	10	[42,271,307,308,309,310,311,312,313,314]
	Papers discussing key management in UIoT networks.	6	[315,316,317,318,319,320]
	Papers discussing information management in UIoT networks.	3	[78,321,322]
	Papers discussing trust management in UIoT networks.	19	[273,275,276,314,323,324,325,326,327,328,329,330,331,332,333,334,335,336,337]

## Data Availability

Not applicable.
